# A one-pot synthesis of AgBr/Ag_3_PO_4_ composite photocatalysts†

**DOI:** 10.1039/d0ra10265b

**Published:** 2021-03-08

**Authors:** Jiahang Su, Yitao Fan, Yu Yan, Tao Liu, Hongshuang Li, Zhenyu Li, Fujiao Song

**Affiliations:** School of Chemical Engineering, Changchun University of Technology Changchun 130012 P. R. China cclzy2001@163.com; Municipal Engineering Northeast Design and Research Institute Co. Ltd Changchun 130112 P. R. China; School of Environmental Science and Engineering, Yancheng Institute of Technology Yancheng 224051 P. R. China song_fj2006@126.com

## Abstract

In this study, an AgBr/Ag_3_PO_4_ (ABAP) photocatalyst has been prepared *via* a facile one-pot anion-exchange method. SEM, XRD, XPS and UV-Vis DRS characterization techniques are carried out to study the structural and physicochemical characteristics of the AgBr/Ag_3_PO_4_ composites. The ABAP photocatalyst exhibited outstanding photocatalytic capability for the photodegradation of rhodamine B (RhB) under visible light irradiation. The optimal ABAP-48% composite displayed the highest photocatalytic activity; a complete degradation was attained in 25 min under visible light irradiation. The excellent stability and reusability of ABAP catalysts were examined by five subsequent runs. A probable degradation mechanism of ABAP composites was carefully surveyed. Furthermore, radical trapping experiments confirmed that the ˙O_2_^−^ radical was the main active species in the photodegradation reaction.

## Abstract 2

In this study, an AgBr/Ag_3_PO_4_ (ABAP) photocatalyst has been prepared *via* a facile one-pot anion-exchange method.

## Graphical abstract

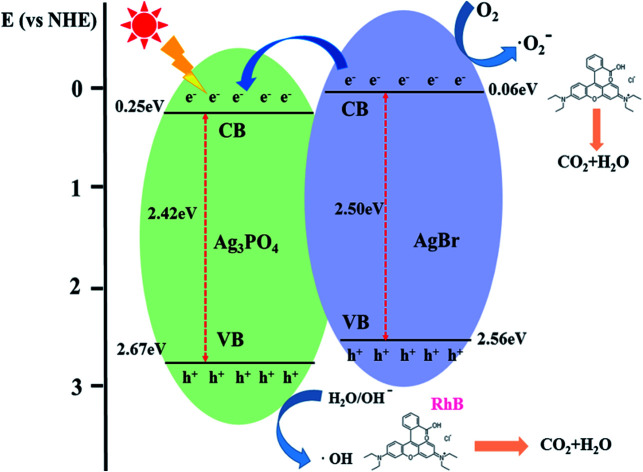

## 1. Introduction

With the rapid development of the textile and paper industries, a great amount of effluent-containing numerous organic dyes has triggered water pollution issues, which has attracted considerable attention of scientists.^1^ The textile effluent consists of suspended solids, grease, lint, surfactants and numerous organic pollutants.^2^ In order to prevent textile waste water from polluting the environment, researchers began to look for ways to treat textile waste water. However, researchers have found that traditional treatment methods, such as physical, chemical and biological methods, could not completely remove complex mixtures and non-biodegradable pollutants from the water.^3^ The semiconductor-based photocatalytic technique has tremendously engaged numerous scholars' attention for purifying air and water due to its advantages of being environmentally friendly and inexpensive.^4^

However, the photocatalytic efficiency of pure Ag_3_PO_4_ is limited by the high recombination rate of its photo-induced electron–hole pairs and poor stability in the photocatalytic process.^5^ So far, numerous strategies have been explored to solve the above-mentioned problems, such as metal doping, construction of semiconductor heterojunctions and carbon-based material recombination. Among these reports, the construction of Ag_3_PO_4_-based heterostructure photocatalysts with other semiconductors is an efficient way to effectively adjust the bandgap structure, and discourage the reorganization of photogenerated electron–hole pairs.^6^ Until now, there are numerous reports about Ag_3_PO_4_-based heterostructure photocatalysts, including M/Ag_3_PO_4_ (M = Pt, Pd, and Au),^7^ Ag@Ag_3_PO_4_@ZnO,^8^ Ag_2_MoO_4_/Ag_3_PO_4_,^9^ Ag_2_S/Ag_3_PO_4_,^10^ Ag/Ag_3_PO_4_/WO_3_,^11^ g-C_3_N_4_/Ag_3_PO_4_,^12^ GO/Ag_3_PO_4_/AgBr,^13^ and nitrogen-doped carbon quantum dots/Ag_3_PO_4_.^14^ Among various Ag-based materials, AgBr is particularly attractive owing to its facile synthesis route, appropriate band gap (∼2.6 eV) as well as excellent photocatalytic activity.^15^ AgBr is regarded as a feasible photocatalyst as it can be excited to generate electron–hole pairs under visible light irradiation. Thus, the weak stability of pure AgBr is a drawback in practical photocatalytic applications. In recent years, numerous AgBr-based heterostructure photocatalysts, such as AgBr/Bi_2_WO_6_,^16^ AgBr/Ag_3_PO_4_,^17^ AgBr/ZnO,^18^ AgBr/MgBi_2_O_6_,^19^ Ag_2_MoO_4_/Ag/AgBr^20^ and g-C_3_N_4_/GO/AgBr,^21^ have been successfully synthesized due to their strong visible light response, high photocatalytic performance, and ability to inhibit electron holes induced by composite light, effectively alleviating the problem of photocorrosion. Among these composite photocatalysts, although AgBr/Ag_3_PO_4_ composite photocatalysts have also been extensively studied, previous studies have reported that the two-step method to synthesize AgBr/Ag_3_PO_4_ photocatalysts has some disadvantages, such as complicated synthesis process, poor photocatalytic activity and low stability of the products.^22,23^ A few studies related to the decoration of AgBr NPs onto the surface of Ag_3_PO_4_ by one-pot method have also been reported. However, the preparation of efficient and environmentally friendly AgBr/Ag_3_PO_4_ photocatalysts with uniformly distributed AgBr NPs on the surface of Ag_3_PO_4_ is crucial to achieve ideal results.

The aim of this project is to prepare a series of ABAP photocatalysts *via* a one-pot anion-exchange method. The performance of the absorption of ABAP photocatalysis with different contents of Br/P was evaluated by the comparing removal rates of RhB. The optical photo-degradation of the ABAP composite in the visible-light range can be highly increased owing to efficient photogenerated electron and hole separation and shifting mechanism. In addition, the possible photodegradation mechanism of the ABAP photocatalyst is also proposed.

## 2. Experiment

### 2.1 Materials and synthesis

Silver nitrate (AgNO_3_), sodium bromide (NaBr) and absolute ethanol were purchased from Beijing Chemical Works. Sodium phosphate monobasic dihydrate (NaH_2_PO_4_), hexamethylene tetramine (HMT), isopropanol (IPA), ammonium oxalate (AO) and *p*-benzoquinone (BQ) were purchased from Sinopharm Chemical Reagent Co., Ltd. All of the regents used in this experiment were of analytical reagent grade without further purification. Ultrapure water utilized in the experiments was obtained from a Milli-Q ultrapure (18.25 MΩ cm) system.

The AgBr/Ag_3_PO_4_ composites were synthesized *via* a facile one pot *in situ* anion-exchange method at room temperature. In a typical synthesis, 0.315 g HMT was added to 40 ml of 0.05 M AgNO_3_ solution under vigorous stirring, and then, the solution became milky white. The mixture was continually stirred for 15 min at room temperature. Subsequently, NaH_2_PO_4_ (0.15 M) was added dropwise to the above solution, and continuously stirred for another 15 min. Afterwards, an NaBr (0.1 M) solution with different volume was added drop by drop to the above solution, and stirred for another 4 h. Finally, the precipitates obtained by centrifugation were then washed several times with ultrapure water and absolute alcohol, dried in an oven at 80 °C for 24 h. The products were named as ABAP-*X*% (*X* is the theoretical molar ratio of Br to P in AgBr/Ag_3_PO_4_ composites). As a reference, pure AgBr or Ag_3_PO_4_ were synthesized *via* the same method without the addition of NaH_2_PO_4_ or NaBr.

### 2.2 Characterization

The morphology and microstructure of the samples were characterized by scanning electron microscopy (SEM) using a model XL30ESEM FEG. The crystalline structure and phase purity of the samples were confirmed *via* X-ray diffraction (XRD) on a Rigaku/Smartlab instrument with the X-ray diffractometer with Cu Kα radiation at a scan rate of 0.01° 2*θ* s^−1^. UV-Vis diffuse reflectance spectra (UV-Vis DRS) were recorded on an Agilent/Cary 5000 using BaSO_4_ as the reference material.

### 2.3 Photocatalytic activity test

RhB was utilized as the simulated dye wastewater to assess the photocatalytic activity of the as-prepared samples. Visible light irradiation was provided by a 500 W xenon lamp (Shanghai Jia Peng Technology Co., Ltd.) equipped with a glass filter (*λ* > 420 nm), which was positioned about 7–10 cm above the solution. In this experiment, the samples were added into a cylindrical quartz glass reactor with 50 ml RhB solution. Before light irradiation, the suspension was stirred for 30 min in dark to ensure the adsorption–desorption equilibrium between the catalysts and RhB solution. In the experimental process, 5 ml of the suspension was taken out at the intervals of the same duration, and immediately separated by centrifugation. Subsequently, the supernatant was monitored using a TU-1901 dual-beam UV-Vis spectrophotometer (Beijing Purkinje General Instrument Co., Ltd.) at the characteristic absorption wavelength (*λ*_max_ = 554 nm) of RhB. The photocatalytic degradation rate could be estimated according to the following eqn (1):1
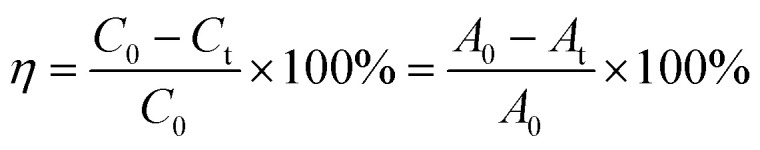
where *C*_0_, *A*_0_ and *C*_t_, *A*_t_ are the initial concentration and ultimate concentration of RhB during the photocatalytic reaction, respectively. The active species trapping experiments of ABAP-*X*% composites were carried out under visible light irradiation for RhB degradation with the addition of isopropanol (IPA), ammonium oxalate (AO) and *p*-benzoquinone (BQ) as scavengers for ˙OH, h^+^ and ˙O_2_^−^.

## 3. Results and discussion

### 3.1 Sample characterization

**Fig. 1:**
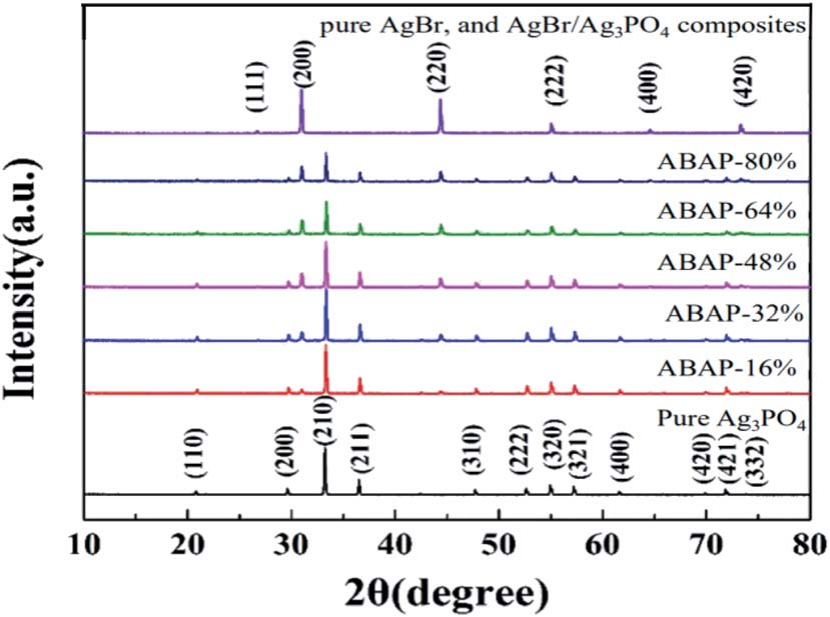
X-ray diffraction patterns of (a) pure Ag_3_PO_4_, (b) ABAP-16%, (c) ABAP-32%, (d) ABAP-48%, (e) ABAP-64%, (f) ABAP-80%, (g) pure AgBr, and AgBr/Ag_3_PO_4_ composites.

XRD was used to validate the crystalline structure of the as-synthesized samples. It can be seen from Fig. 1 that all of the identified peaks in the XRD pattern could be attributed to the body-centered cubic phase Ag_3_PO_4_ (JCPDS no. 06-0505), indicating that Ag_3_PO_4_ had a body-centered cubic structure. The strong and sharp peaks of Ag_3_PO_4_ nanoparticles indicated its high crystallinity. Close to the JCPDS cards of pristine AgBr (no. 06-0438) (Fig. 1(g)), the characteristic diffraction peaks belong to the face-centered cubic structure. No impurity peaks were detected in the sample, which showed that the samples were pure AgBr phase. Compared to pure Ag_3_PO_4_ and AgBr, the peak intensities of ABAP composites were obviously weaker. It seemed that a part of the XRD signal of Ag_3_PO_4_ particles was hindered by the surface-coated AgBr, which contributed to the decrease in the intensity of XRD diffraction peaks. It is expected that the XRD pattern of the ABAP composites clearly matched with the polycrystalline structures of AgBr and Ag_3_PO_4_, suggesting that AgBr and Ag_3_PO_4_ maintained pure phase, and there were no impurities in the ABAP composites. It is indicated that the ABAP composites have been successfully prepared.

**Fig. 2:**
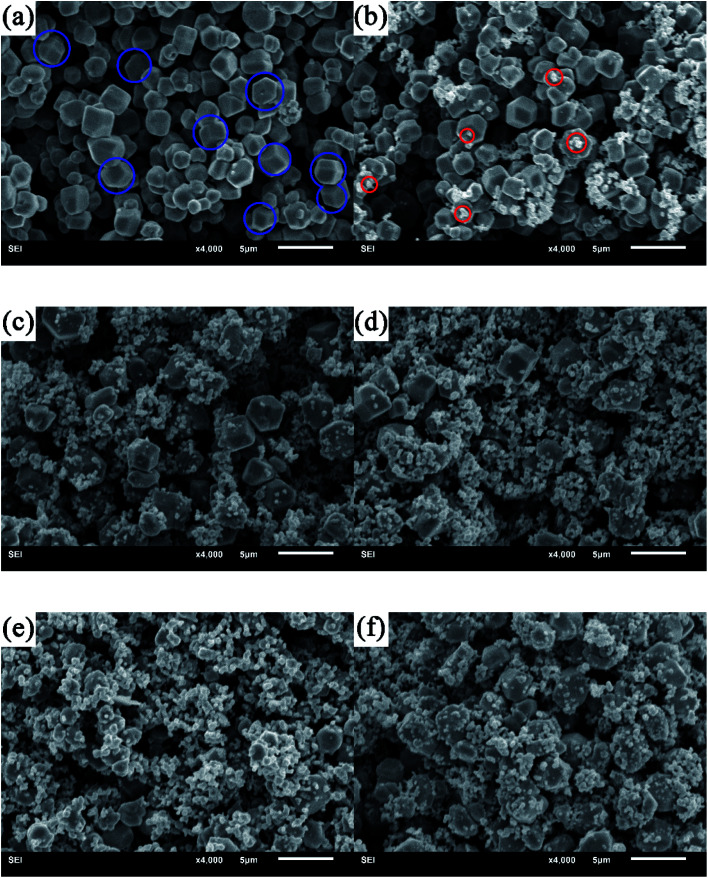
Typical SEM images of (a) pure Ag_3_PO_4_, and AgBr/Ag_3_PO_4_ composites, (b) ABAP-16%, (c) ABAP-32%, (d) ABAP-48%, (e) ABAP-64%, (f) ABAP-80%.

The comparative morphology of the as-synthesized Ag_3_PO_4_ and ABAP composites with different concentrations of Br/P were characterized by SEM images shown in Fig. 2. It can be observed that the polyhedral structure particle is the Ag_3_PO_4_ material in Fig. 2(a) (blue circle), and the average diameter of Ag_3_PO_4_ is 1 μm. Furthermore, we can clearly observe that the surface of Ag_3_PO_4_ is very smooth and clean. The pure AgBr nanoparticles are irregular aggregates composed of grains with diameters of 2 to 7 nm (Fig. S1†). It can be seen in Fig. 2(b) that there are a few cluster particles coated on the smooth surface of Ag_3_PO_4_ (red circle), which are irregularly aggregated AgBr nanoparticles. After coating with an AgBr layer, we can obviously see that AgBr nanoparticles were attached closely to the surface of Ag_3_PO_4_, which is favourable to the building of heterojunction with different concentrations of Br/P between Ag_3_PO_4_ and AgBr (Fig. 2(c)–(f)). From the SEM images, we can see that ABAP composites have nearly the same morphology as that of Ag_3_PO_4_. During the one-pot preparation of ABAP-48%, Ag_3_PO_4_ crystals were first synthesized. Then, AgBr was assembled over the outer layer of Ag_3_PO_4_. This result is in good agreement with the XRD observations discussed above.

**Fig. 3:**
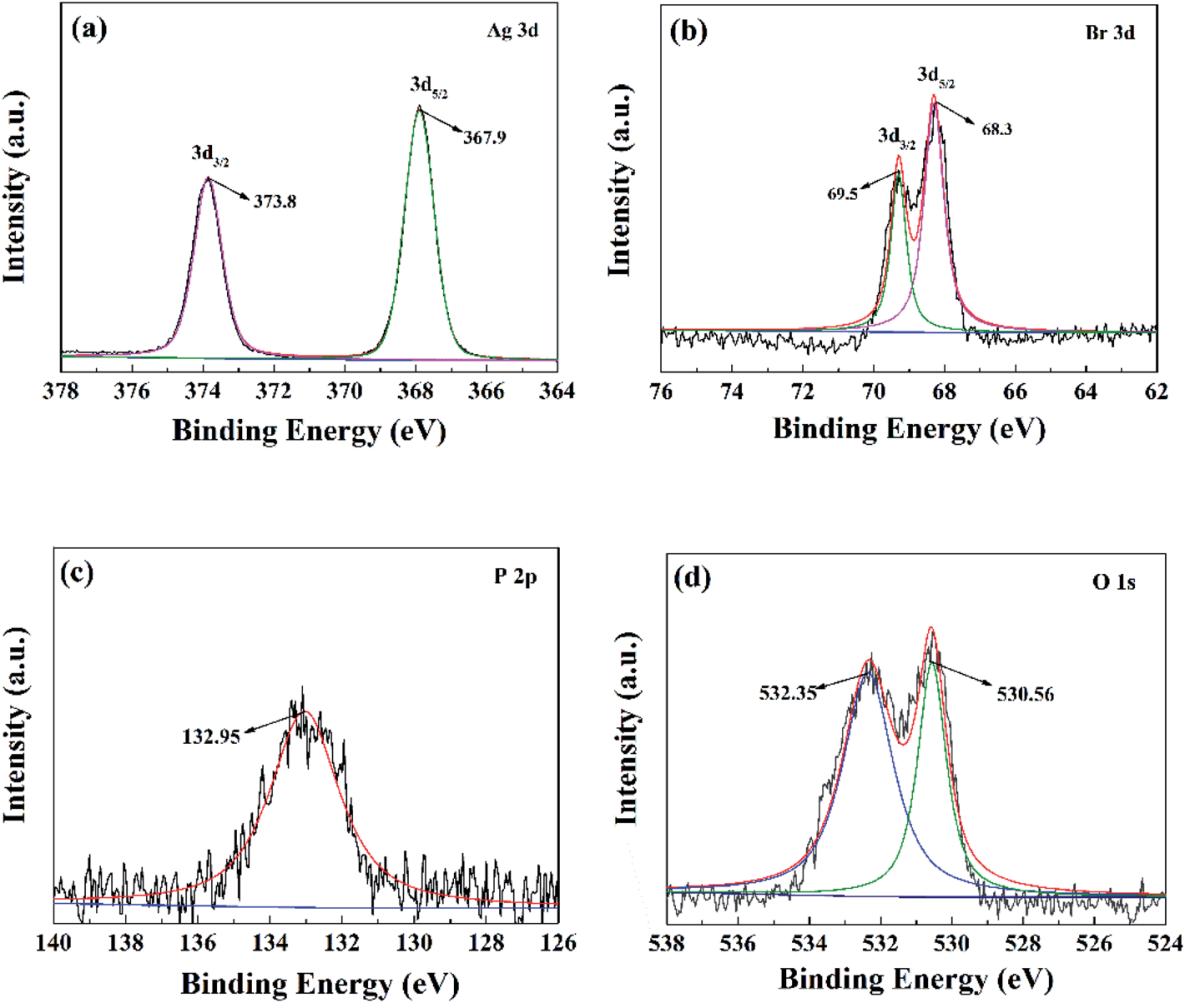
XPS spectra of (a) Ag 3d, (b) Br 3d, (c) P 2p and (d) O 1s of the ABAP-48% composites.

**Fig. 4:**
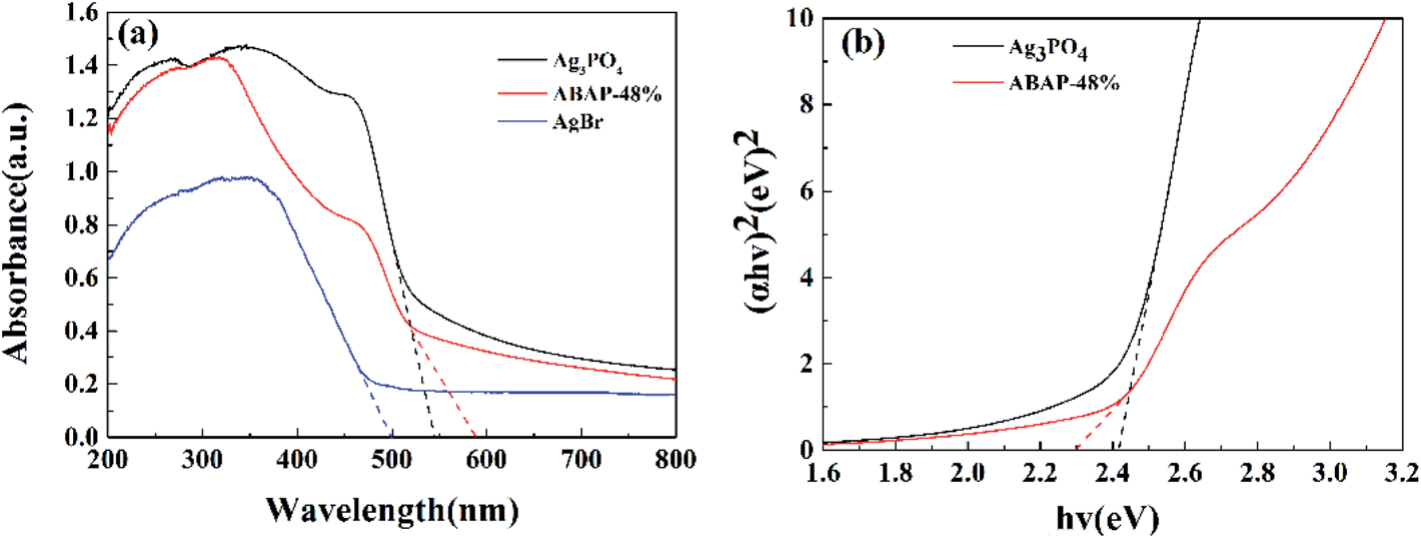
UV-Vis diffuse reflectance spectra of (a): Ag_3_PO_4_, AgBr, ABAP-48%; (b) shows plot of (*αhν*)^2^*versus* energy (*hν*) Ag_3_PO_4_ for and ABAP-48%.

The elemental composition and chemical valance state of the notable ABAP-48% were investigated by XPS, and the XPS results are illustrated in Fig. 3. The survey spectrum of ABAP-48% can clearly illustrate the presence of Ag, P, O, and Br in Fig. S2,† which originated from Ag_3_PO_4_ and AgBr.^24–26^ The noticeable peaks are at 373.8 eV and 367.9 eV of Ag 3d in Fig. 3(a), which correspond to Ag 3d_3/2_ and Ag 3d_5/2_, indicating the presence Ag^+^ in Ag_3_PO_4_ or AgBr. The high-resolution spectrum of Br 3d in Fig. 3(b) demonstrates two peaks at 69.5 and 68.3 eV, which correspond to the binding energies of Br 3d_3/2_ and Br 3d_3/2_.^27^ The peak located at 132.95 eV in the P 2p spectrum in Fig. 4(c) elucidates the presence of P^5+^ in PO_4_^3−^. The strong peak in the O 1s spectrum of the sample in Fig. 3(d) at 530.6 eV is ascribed to the oxygen in the Ag_3_PO_4_ crystal lattice, and the weak peak at 532.4 eV is due to the chemisorbed H_2_O or OH^−^ on the surface of the samples.^28–30^ On the basis of the above-mentioned results, the AgBr nanoparticles were successfully attached to the Ag_3_PO_4_ surface, in accordance with the XRD and SEM results.

In order to study the light absorption characteristics of the heterojunction composite photocatalytic materials, the UV-Visible diffuse reflection absorption curves of the samples were compared. It can be seen from Fig. 4(a) that Ag_3_PO_4_ shows a strong visible light absorption edge located at 550 nm, which is similar to the previous reports.^31,32^ The visible light absorption intensity of ABAP-48% is significantly higher than that of pure AgBr and pure Ag_3_PO_4_. The absorption edges of ABAP-48% composite photocatalyst reflect an obvious redshift to 590 nm compared to that of Ag_3_PO_4_ and AgBr. This obvious redshift of the absorption edge of the as-prepared sample is ascribed to the excellent sensitization effect of AgBr, which affirms that a large number of photo-induced carriers could be excited by more visible light. This result suggests that Ag_3_PO_4_ and AgBr have been composited successfully. Simultaneously, the results also show that the composite photocatalyst has a stronger absorption capacity for visible light, which means the increase in the efficient response to sunlight, and which is advantageous for the photocatalytic activity.

The plots of (*αhν*)^*n*/2^*versus* energy for Ag_3_PO_4_ and ABAP-48% are shown in Fig. 4(b). The *E*_VB_ and *E*_CB_ of Ag_3_PO_4_ and AgBr can be respectively calculated according to the experiential formula (2) and (3) listed below:

2 *E*_VB_ = *X* − *E*_0_ + 1/2*E*_g_

3 *E*_CB_ = *X* − *E*_0_ − 1/2*E*_g_

where *X* is the absolute electronegativity of the semiconductor, and *E*_VB_ and *E*_CB_ are the potential of VB and CB, respectively. The value of *E*_0_ is 4.5 eV and *E*_g_ is the band gap energy of the semiconductor. The *X* values of AgBr and Ag_3_PO_4_ are 5.81 eV and 5.96 eV, respectively. According to the UV-Vis DRS, the reckoned band gap energies of the as-synthesized pure Ag_3_PO_4_ and AgBr were detected to be 2.42 and 2.50 eV, respectively. Based on the above experimental formula, the highest part of the VB and the lowest part of the CB of Ag_3_PO_4_ are 2.67 eV and 0.25 eV, respectively. Simultaneously, the VB and CB of AgBr are detected to be 2.55 eV and 0.07 eV, respectively. In conclusion, the results of XRD, SEM and UV-Vis diffuse spectra proved the formation of the Ag_3_PO_4_ heterojunction in ABAP nanoparticles, which may demonstrate high photocatalytic efficiency.

### 3.2 Photocatalytic performance

**Fig. 5:**
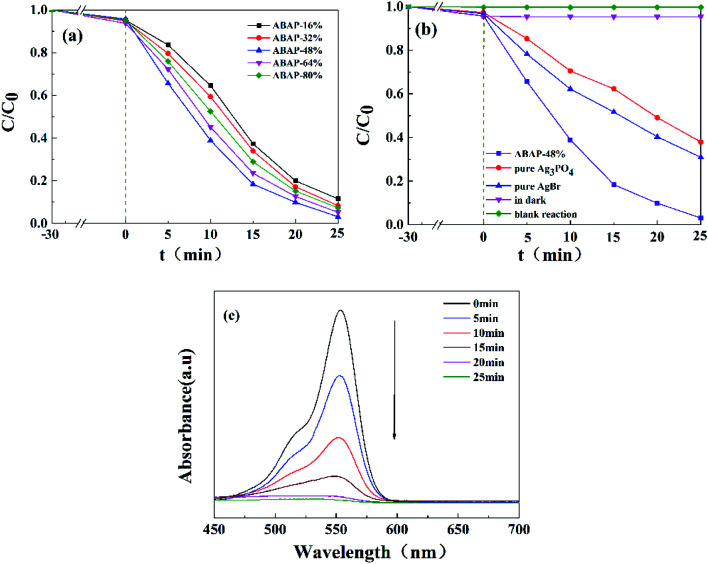
(a) Photocatalytic activities of ABAP-*X*% for RhB degradation under visible-light (*λ* > 420 nm); (b) photocatalytic activities of numerous photocatalysts under visible-light (*λ* > 420 nm); (c) UV-Vis spectral absorption changes of RhB solution photodegraded over the ABAP-48% composite under visible light irradiation.

The photocatalytic activities of pure Ag_3_PO_4_, pure AgBr and ABAP composites were determined by the degradation of RhB under visible light irradiation. The reusability tests were different from the original photocatalytic tests (Fig. 5); it was not necessary to detect every sample with a 5 min interval, only to compare the results after 25 min. After each test, the used sample was centrifuged and washed with deionized water three times, and dried for the next recycled photoactivity test. Before the light irradiation, the adsorption capability was primarily assessed in the absence of light. A liquid mixture of RhB and the resulting samples was vigorously stirred without irradiation for 0.5 h to achieve the adsorption–desorption equilibrium. It is shown in Fig. 5(a) that the concentration of RhB has only a minor decline without photocatalyst or without visible light irradiation, which indicated that the blank degradation was inappreciable. Under visible light illumination for 25 min, about 62.1% and 69.1% of original RhB was eliminated by pure Ag_3_PO_4_ and pure AgBr, respectively. It is obviously observed that the photocatalytic degradation capabilities of RhB are approximately 88.5%, 91.7%, 96.9%, 94.8% and 92.7% for ABAP-16%, ABAP-32%, ABAP-48%, ABAP-64%, ABAP-80%, respectively, in the presence of visible light irradiation (*λ* > 400) for 25 min. The ABAP-48% sample displayed a much higher performance than the other different constant ABAP-X% composites. However, at a higher theoretical molar ratio of Br relative to P (>48%), the photocatalytic activity decreased, indicating that a few active sites were covered with cohered AgBr coated on the surface of Ag_3_PO_4_. It suggested that the photocatalytic activities of the samples depended on the mass ratio of Br relative to P, demonstrating that AgBr played an important role in the synergic effects between AgBr and Ag_3_PO_4_. As shown in Fig. 5(b), the intensity of the absorption spectra of RhB decreased by the ABAP-48% composite in the process of photocatalytic degradation. As the illumination time prolonged, the absorption peak intensity of RhB decreased step by step, and almost 100% of RhB degraded after illumination for 25 min.

**Fig. 6:**
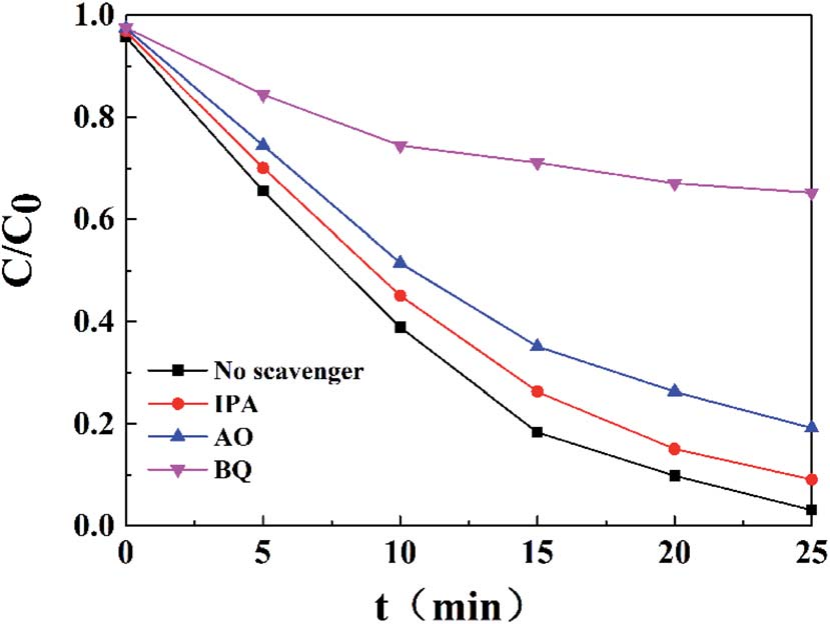
Effects of different scavengers on the degradation of RhB over ABAP-48% under visible light irradiation.

**Fig. 7:**
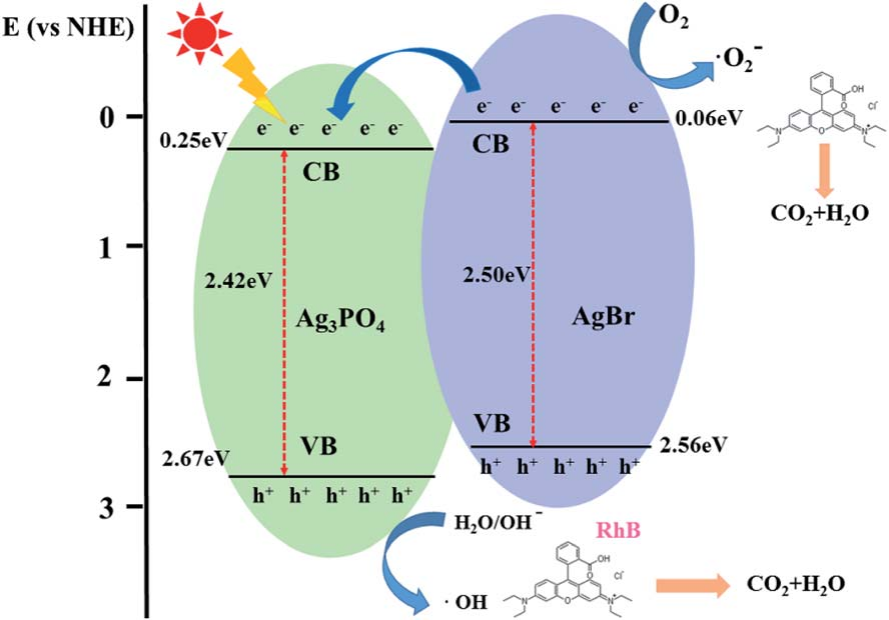
Schematic showing the energy band structure and electron–hole pair separation in the ABAP-48% heterostructure under visible light irradiation.

To further investigate the major role of ABAP composites in the photocatalytic activity, a series of radical-trapping experiments were conducted by adding different scavengers. In general, several reactive intermediate species, such as h^+^, ˙OH, and ˙O_2_^−^, are produced during the photocatalytic process. Hence, we used IPA, AO and BQ as the active scavengers for ˙OH, h^+^ and ˙O_2_^−^, respectively. As shown in Fig. 6, it can be observed that only a minor change was caused when IPA and AO were added to the reaction system for the photocatalytic degradation of RhB in the presence of ABAP-48% under visible light irradiation, which suggested that ˙OH and h^+^ were not the crucial active species in this reaction system. Conversely, when BQ was added, the photocatalytic activity was greatly suppressed, and the photo-degradation rate dropped to 25% compared to the no scavenger reaction under the same conditions, which indicated that ˙O_2_^−^ played the main role in the reaction process (Fig. 7).

### 3.3 The possible mechanism of photocatalytic degradation

**Fig. 8:**
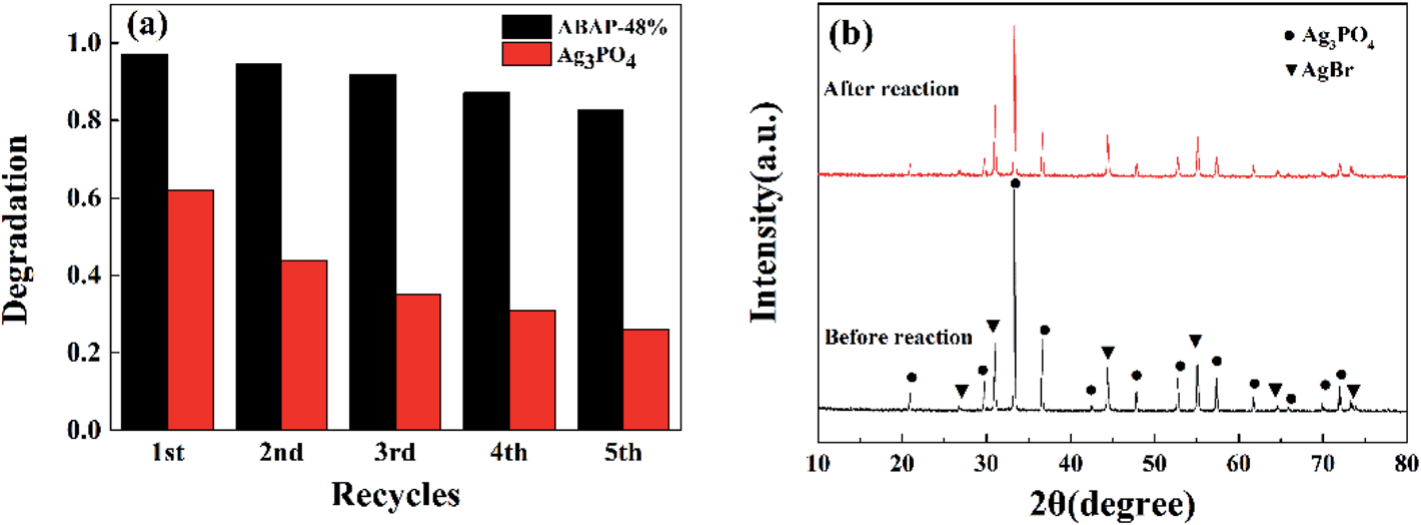
(a) The cycling runs in the photocatalytic degradation of RhB over ABAP-48% and pure Ag_3_PO_4_ under visible light irradiation; (b) XRD patterns of ABAP-48% after the photocatalytic reaction.

It is known that the efficient separation and transfer of photo-induced carriers at the heterojunction interfaces is an important strategy to improve the photocatalytic activity of composite photocatalysts.^28^ Based on the above-mentioned results, a feasible photocatalytic mechanism of the as-synthesized ABAP composite under visible light irradiation is illustrated in Fig. 8. In this photocatalyst system, due to the irradiation of visible light, AgBr nanoparticles deposited on the surface of Ag_3_PO_4_ nanoparticles can successfully reduce the solubility of Ag_3_PO_4_ in the aqueous solution since AgBr nano-particles can transfer or store electrons. Under visible light irradiation, both Ag_3_PO_4_ and AgBr are induced as well as the photo-generated electron and hole pairs are distributed in their CB and VB, respectively. However, the band edge potential position of the Ag_3_PO_4_ and ABAP composites had a significant influence on researching the process of photo-generated electron and hole pairs in the heterojunction. This made it difficult for the adsorbed water to be converted into ˙OH radicals by light-induced holes. For the ABAP heterojunction photocatalysts, the CB and VB potentials of AgBr are more negative than those of the pristine Ag_3_PO_4_. The above-mentioned result shows that photo-induced electrons in AgBr immediately transferred to the CB of the Ag_3_PO_4_ particles. The reaction of photo-induced electrons on the CB of Ag_3_PO_4_ with molecular oxygen could produce superoxide radicals (˙O_2_^−^), which could engage in the photocatalytic decomposition process of RhB. It promoted the effective separation of photoexcited electron–hole pairs, and decreased the probability of electron–hole recombination, so RhB could be degraded more efficiently after the addition of AgBr. It suggested that the assembling of Ag_3_PO_4_ and AgBr is beneficial to enhance the transfer of photo-generated electrons–hole pairs, decreasing the possibility of the recombination of photo-excited carriers, and accelerating the generation of more ˙O_2_^−^. Thus, the photocatalytic activity of pristine particles could be tremendously enhanced after the addition of AgBr.

### 3.4 The recyclability study

In addition to the photocatalytic activity, another significant factors for the practical application of composite photocatalysts are stability and reusability. Under visible light irradiation, the as-prepared ABAP composites were also investigated by the circulating runs in the photocatalytic degradation of RhB to evaluate the stability. As shown in Fig. 8(a), the photocatalytic performance of pure Ag_3_PO_4_ showed a notable loss after five cycles, while that of the ABAP-48% composite remained at 82%, which indicated that the AgBr nanoparticles anchoring on the surface of Ag_3_PO_4_ nanoparticles can obviously decrease the solubility of Ag_3_PO_4_ in the aqueous solution. Thus, the structural stability of ABAP composites considerably increased in the photocatalytic processes. The crystalline structures of the ABAP-48% nanocomposite were investigated before and after reactions to assess the structural stability. Fig. 8(b) displays the XRD patterns of ABAP-48% before and after circulating experiments. No distinct structure or integrity changes of the photocatalyst can be observed in the XRD pattern, which indicates that AgBr enhances the stability of the Ag_3_PO_4_ photocatalyst. In short, a certain amount of AgBr attached to the surface of the Ag_3_PO_4_ nanoparticles can evidently enhance the photocatalytic activity and stability of the ABAP composites compared to the original Ag_3_PO_4_. This result shows that the composites can be widely used in the photocatalytic degradation of numerous organic pollutants in waste water as a series of hopeful photocatalysts.

### 3.5 Determination of TOC after waste liquid degradation

**Fig. 9:**
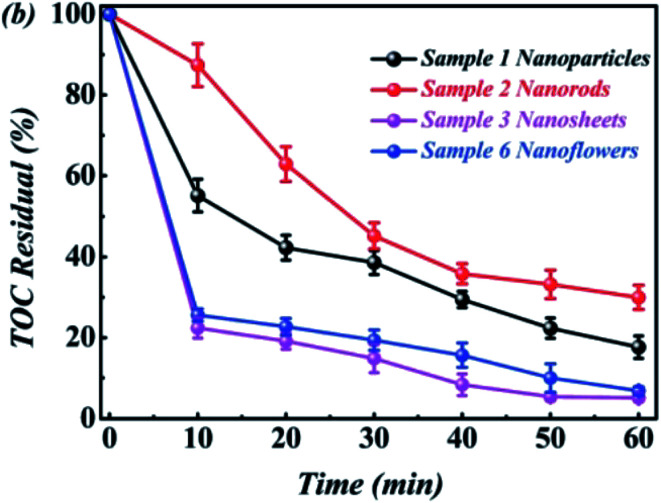
The TOC removal of RhB degradation products.

The total organic carbon (TOC) analysis of the RhB solution was carried out using a WTW's photoLab S12 analyzer. Taking sample ABAP-48% as an example, Fig. 9 shows the TOC removal of RhB degradation products. About 99% of TOC was removed within 40 min in the presence of ABAP-48%, demonstrating that RhB was oxidized by oxidative radicals to CO_2_, H_2_O, and NO_3_^−^, indicating that the photocatalytic RhB oxidation process did not produce secondary harmful species.

## 4. Conclusion

In summary, in this study, different contents of AgBr nanoparticles deposited on Ag_3_PO_4_ polyhedral structure particles were successfully prepared by the anion-exchange of Br^−^ with PO_4_^3−^. AgBr nanoparticles with sizes of less than 7 nm were evenly anchored on the surface of Ag_3_PO_4_ polyhedral structure particles. The ABAP-48% composites exhibited a much higher activity than original Ag_3_PO_4_ and AgBr for the decomposition of RhB, which simulated dyes in waste water. This could be profited from the stronger response to visible light and photo-induced electron and hole separation and transfer mechanism. It is suggested that ˙O_2_^−^ played an important role in the photocatalytic degradation of RhB by ABAP composite photocatalysts in the presence of visible light. Moreover, the ABAP-48% composites showed an excellent photocatalytic activity after five recycling runs, which demonstrated the high stability. Consequently, ABAP composites synthesized by the one pot method had obvious advantages in the waste water treatment and environmental restoration under an irradiation with visible light.

## Conflicts of interest

We declare that we have no financial and personal relationships with other people or organizations that can inappropriately influence our work; there is no professional or other personal interest of any nature or kind in any product, service and/or company that could be construed as influencing the position presented in, or the review of, the manuscript entitled, “One-pot synthesis of AgBr/Ag_3_PO_4_ composite photocatalyst for enhancing visible photocatalytic degradation of RhB”.

## Supplementary Material

RA-011-D0RA10265B-s001
