# Supplementary material for: A one-pot synthesis of AgBr/Ag3PO4 composite photocatalysts
Source: RSC Adv. 2021 Mar 8;11(17):9865–73. doi: 10.1039/d0ra10265b (PMC8695699; doi:10.1039/d0ra10265b)
Supplement: RA-011-D0RA10265B-s001 [file RA-011-D0RA10265B-s001.pdf]

Electronic Supplementary Material (ESI) for RSC Advances.
This journal is © The Royal Society of Chemistry 2021

Supporting Information

One-pot synthesis of $AgBr/Ag_3PO_4$ composite photocatalyst

Yitao Fan[1]， Tao Liu[1] , Hongshuang Li[1], Yu Yan[2] ,Zhenyu Li[1]*

1 School of Chemical Engineering, Changchun University of Technology, Changchun 130012, P. R. China.

2 Municipal Engineering Northeast Design and Research Institute Co.LTD,Changchun 130112,P. China.

*Corresponding author: A/Prof. Zhenyu Li. cclzy2001@163.com

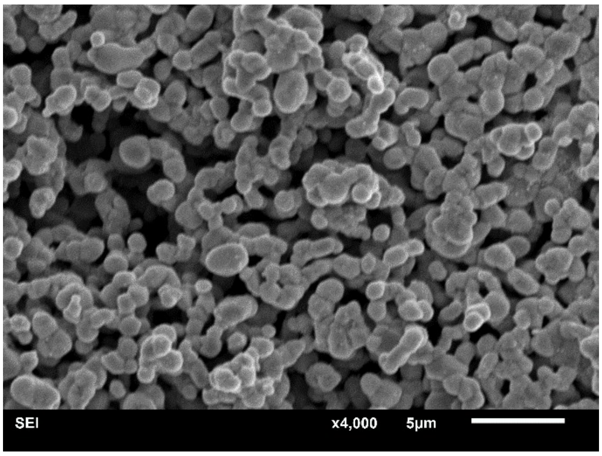

Figure S1 SEM images of the AgBr nanoparticles.

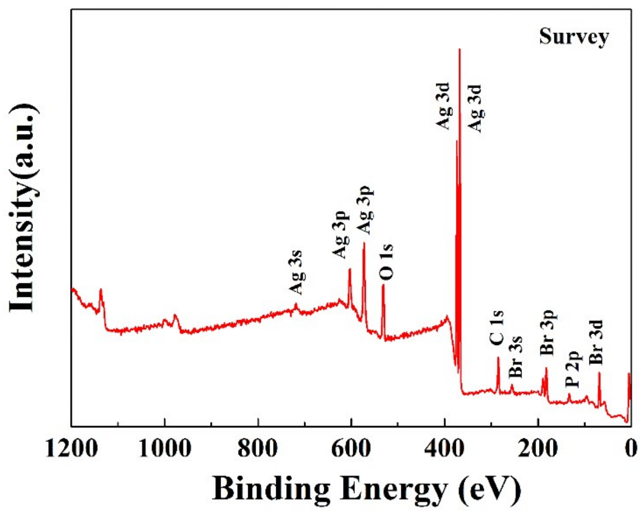

Figure S2 XPS survey spectrum of ABAP-48%
